# A novel variant in *NSUN2* causes intellectual disability in a Chinese family

**DOI:** 10.1186/s12920-024-01883-x

**Published:** 2024-04-20

**Authors:** Qi Yang, Qiang Zhang, Zailong Qin, Shang Yi, Jingsi Luo

**Affiliations:** 1 Guangxi Key Laboratory of Birth Defects Research and Prevention, Guangxi Key Laboratory of Reproductive Health and Birth Defects Prevention, Maternal and Child Health Hospital of Guangxi Zhuang Autonomous Region, No. 59, Xiangzhu Road, Nanning, China; 2 Department of Genetic and Metabolic Central Laboratory, Maternal and Child Health Hospital of Guangxi Zhuang Autonomous Region, Nanning, China

**Keywords:** *NSUN2* gene, Intellectual disability, Novel variant, Whole exome sequencing, Genotype-phenotype correlation

## Abstract

NSUN2-intellectual disability syndrome, also known as intellectual disability type 5 (MRT5), is an autosomal recessive disorder that is characterized by intellectual disability (ID), postnatal growth retardation, dysmorphic facies, microcephaly, short stature, developmental delay, language impairment and other congenital abnormalities. The disease is caused by mutations in the *NSUN2* gene, which encodes a tRNA cytosine methyltransferase that has an important role in spindle assembly during mitosis and chromosome segregation. In this study, we recruited a family that had two individuals with ID. Whole exome sequencing was performed to identify a homozygous frameshift variant (c.1171_1175delACCAT(p.Thr391fs*18*)) in *NSUN2* (NM_017755.5) in the proband. The varint was confirmed as segregating in his affected brother and his parents by Sanger sequencing. The individuals that we described showed a similar dysmorphology profile to that associated with MRT5. To analyze the correlations between genotypes of *NSUN2* and phenotypes of individuals with ID, we examined 17 variants and the associated phenotypes from 32 ID individuals in current and previous studies. We concluded that mutations in *NSUN2* cause a wide range of phenotypic defects. Although some clinical manifestations were highly variable, the core phenotypes associated with NSUN2 mutations were dysmorphic facies, microcephaly, short stature, ID, growth restriction, language impairment, hypotonia and delayed puberty. Our study expands the genetic spectrum of *NSUN2* mutations and helps to further define the genotype-phenotype correlations in MRT5.

**Supplementary Information:**

The online version contains supplementary material available at 10.1186/s12920-024-01883-x.

## Introduction

Intellectual disability type 5 (MRT5, MIM 611091) is a very rare autosomal recessive disorder that is characterized by varying degrees of intellectual disability (ID), facial dysmorphism, microcephaly, short stature, growth restriction, language impairment, hypotonia and delayed puberty [1]. It is caused by homozygous or compound heterozygous mutations in the *NSUN2* gene (NM_017755.5, MIM 610916). This gene is located on chromosome 5p15.31 and encodes the NOP2/Sun transfer RNA methyltransferase family member 2. It influences the level or function of tRNA, mRNA and non-coding RNA by introducing 5-methylcytosine [2, 3]. In addition, it is involved in biological processes such as spindle assembly, chromosome segregation, regulation of cell proliferation and division [4, 5]. The *NSUN2* gene is highly conserved from archaea to eukaryotes, and is expressed in many organs in humans [6]. The above information implies that a functionally impaired *NSUN2* gene would lead to various abnormal phenotypes.

**Fig. 1:**
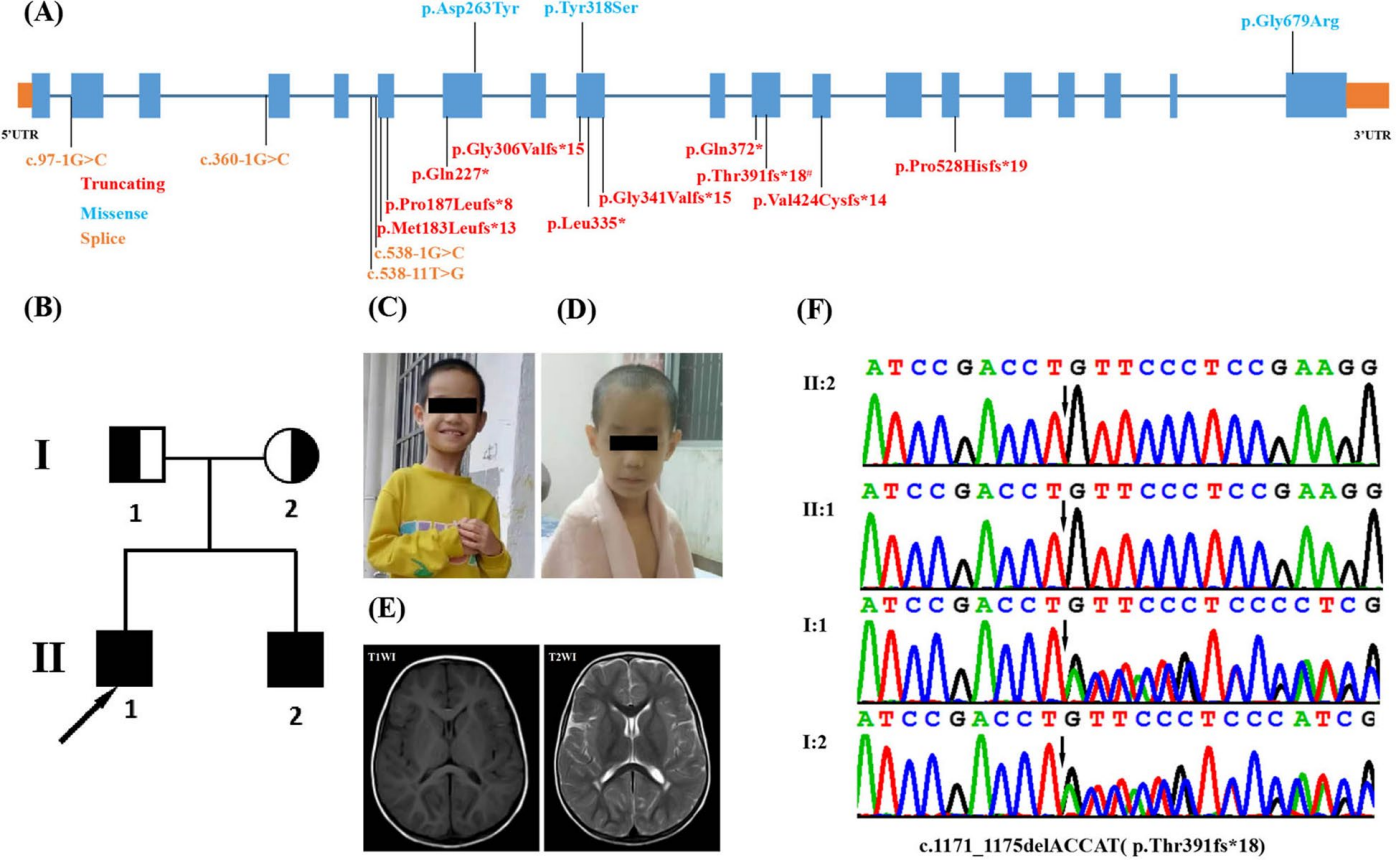
Clinical and genetic features. **A** Distribution of all NSUN2 pathogenic variants. All 17 reported variants are shown (bold = previously reported; # = recurrent variant). **B** Family pedigree shows two affected offspring from nonconsanguineous parents. **C** Facial appearance of patient 1 (II-1) at 9 years old, showing upper eyelid ptosisptosis, long palpebral, fissures high posterior hairline and smooth philtrum. **D** Facial appearance of patient 2 (II-2) at 6 years old, showing long face, long palpebral fissures, high posterior hairline and long philtrum. **E** Axial slices of T1weighted images (T1WI) and T2WI, acquired at 5 years in patient 2, showing hypomyelination. **F** DNA sequence chromatograms from Sanger sequencing of *NSUN2*, showing a homozygous frameshift mutation c.1171_1175delACCAT(p.Thr391fs*18) in the proband. Sanger sequencing further revealed that his affected brother was homozygous for the same mutation and that his parents were heterozygous

Recently, 16 loss-of-function (LoF) *NSUN2* mutations were identified in 30 patients with intellectual disability/developmental delay (ID/DD) and growth retardation. These mutations included seven frameshift variants, three nonsense variants, two missense variants and three splicing variants [1, 7–17] (Fig. 1A). Mutations in NSUN2 lead to a wide range of phenotypic defects. They include microcephaly, ID, short stature, speech delay, motor delay, delayed puberty, and dysmorphic facies, such as hypertelorism, full upper lip, long face, long nose, high nasal bridge and short philtrum, infertility, chronic nephritis, hearing impairment, seizures, cerebellar atrophy, hypomyelination, dysplastic corpus callosum and simplified gyral patterning of the frontal lobe. The severity of the phenotypes caused by mutations in the *NSUN2* gene remains to be fully explored. Therefore, additional reports on *NSUN2* mutations and their phenotypes will be helpful for understanding this condition. Here, we investigated a previously unreported, homozygous frameshift variant (c.1171_1175delACCAT(p.Thr391fs*18*)) in *NSUN2*, in a Chinese family with two affected individuals (Fig. 1B, C). This mutation was associated with autosomal recessive intellectual developmental disorder type 5. We also described the relevant clinical profiles of the individuals and reviewed the overall phenotypic deficits associated with MRT5.

## Materials and methods

### Editorial policies and ethical considerations

A Chinese family was referred to Guangxi Maternal and Child Health Hospital for ID/ DD and epilepsy testing. Genetic testing was requested. The project was approved by the Medical Ethics Committee of the Maternal and Child Health Hospital of Guangxi Autonomous Region and detailed, written, informed consent was obtained from the parents for publication of the patient’s pertinent images in this paper.

### Whole exome sequencing and Sanger sequencing

The Lab-Aid DNA kit (Zeesan Biotech Co., Ltd., Xiamen, China) was used to obtain genomic DNA from peripheral blood samples from all family members, in accordance with the manufacturer's protocol. The DNA concentration and purity were determined by a NanoDrop 1000 spectrophotometer (Thermo Scientific). Whole exome capture was performed using the Agilent SureSelect Human Exon V6 kit (Agilent Technologies, Santa Clara, CA, USA) and the Illumina HiSeq 2000 platform (Illumina Biotechnology, San Diego, CA, USA) was used for sequencing. A read depth of 120× and a sequencing depth > 20× were used for 98% of the targeted regions. The reads were aligned to the human genome reference (UCSC GRCh37/hg19), using Burrows-Wheeler Aligner (BWA-MEM, version 0.7.10). Variant detection was performed using the Genome Analysis Toolkit (GATK) 3.4 (GATK, www.broadinstitute.org/gatk). TGex software (LifeMap Sciences, Alameda, CA, USA) was used to annotate and classify the variants.

Candidate variants were filtered based on the following filtering criteria: (a) a minor allele frequency below 0.1% for variants in the public databases (e.g., 1000 Genomes Project, Exome Sequencing Project and ExAC) and our in-house databases, (b) exonic variants and intronic variants (located in the splice site region, within 10 bp of the splice site) and (c) LoF alleles or damaging missense variants, predicted by functional prediction tools (e.g., PROVEAN, PolyPhen2, CADD and MutationTaster).

Four tools, PROVEAN (http://provean.jcvi.org/), PolyPhen2 (http://genetics.bwh.harvard.edu/pph2/), CADD (https://cadd.gs.washington.edu/snv) and MutationTaster (http://www.mutationtaster.org/) were used to predict the effect of the candidate variants, which were then confirmed by Sanger sequencing of the proband, his affected brother and other unaffected family members. Validation was performed by PCR and primers were designed to amplify the *NSUN2* mutation (NM_017755.5: c.1171_1175delACCAT(p.Thr391fs*18)). The primer sequences were 5'-TGCTAGTAATCTGGAATGTACCC-3′ and 5′-ACCCCAACAAGACTCACCAG-3′. The product length was 248 bp. The pathogenicity of the candidate *NSUN2* variant was classified in accordance with the American College of Medical Genetics and Genomics/Association for Molecular Pathology (ACMG/AMP) guidelines [18].

## Results

### Clinical phenotype

Patient 1 (II:1) was a 9-year-old male who was the first child of a physically healthy, non-consanguineous Chinese couple. He had a birth weight of 2.8 kg and a gestational age of 39 weeks. His development was globally delayed. He could sit at ten months and walk at three years of age but was not able to speak. A physical examination showed that he had severe short stature (height: 110 cm, < −4 SD), low body weight (15 kg) and microcephaly (head circumference of 47 cm, < −3 SD). Mild facial dysmorphic features were observed and included right upper eyelid ptosis, long palpebral, fissures high in the posterior hairline and a smooth philtrum. The Wechsler Intelligence Scale for Children was used at nine years of age and the patient’s full scale IQ was 40. He had his first seizure at four years and two months of age. The type of seizure was generalized tonic-clonic seizure (GTCS), with a frequency of two to four times a day. Each seizure lasted for approximately ten seconds to one minute. The electroencephalogram (EEG) results were abnormal at four years and two months of age, showing a sharp wave, spike wave and slow wave in the right occipital and left middle temporal regions. The seizures were controlled with valproate (VPA). The initial brain MRI, at four years and five months of age, was normal. Hearing problems were not observed.

Patient 2 (II:2) was the younger brother of patient 1. He was born prematurely at 34 weeks, with a birth weight of 1850 g. He was placed in an incubator for the first month of life, due to premature birth, neonatal hypotonia, feeding difficulties and mild respiratory distress. His milestone development was delayed and he started to sit without support at 11 to 12 months, to crawl at 17 months and to walk at 53 months. The patient was unable to speak. The Gesell Developmental Quotient Score was 32, at two years and three months of age. His most recent examination, at the age of six, revealed postnatal growth retardation, short stature (height: 100 cm, < −3 SD), microcephaly (head circumference of 46.1 cm, < −3.4 SD) and dysmorphic facial features, which included a long face, long palpebral fissures, high posterior hairline and long philtrum. A brain MRI was performed at five years and revealed hypomyelination (Fig. 1D). He also had moderate hearing impairment.

### Molecular analysis

To identify the potential genetic mutation that led to the ID in this family, whole exome sequencing (WES) was performed on the proband. This yielded 5.3 Gb of data, with 99.7% coverage of the target region and 98.8% of targets covered over 20 times. A total of 25,835 single nucleotide variants (SNV) or insertion/deletion (indel) variants were identified in coding regions and splice sites (10 bp from the splicing junction). Data filtering excluded low-confidence variants and common variants with allele frequencies (AF) > 1% in the public databases (e.g., 1000 Genomes Project, Exome Sequencing Project and ExAC) and our in-house databases. We also excluded likely benign and benign variants, which included synonymous and harmless missense variants predicted by PROVEAN, PolyPhen2, CADD and MutationTaster. Five hundred and twenty-one variants remained. Clinical symptoms of seizures, ID, global DD, short stature, low body weight and microcephaly were then used as filtering indices to analyze candidate variants. Using TGex software (LifeMap Sciences, United States), nine candidate variants in eight genes (*NSUN2*, *KANSL1*, *SLC1A2*, *SPTLC1*, *NOTCH1*, *TTN*, *ADGRV1*, *ATAD3A*) were matched with known phenotypes and subsequently extracted. As a result, a homozygous frameshift variant in exon eleven of *NSUN2* (NM_017755.5: c.1171_1175delACCAT(p.Thr391fs*18)) was identified in both patients. This variant was validated by Sanger sequencing and both parents were shown to carry the variant in heterozygous form (Fig. 1F).

The *NSUN2* c.1171_1175delACCAT(p.Thr391fs*18) variant is a novel variant, that is not found in the exome database, Human Gene Mutation Database, 1000 Genomes Database, ClinVar database, Exome Sequencing Project, dbSNP, ExAC or gnomAD. It is located in the eleventh exon of the *NSUN2* gene and causes a premature termination codon, which may activate the nonsense-mediated mRNA decay (NMD) pathway. This will result in a marked decrease in the level of *NSUN2* mRNA expression and a loss of function. The functional prediction for c.1171_1175delACCAT(p.Thr391fs*18) is ‘disease-causing’, as determined by MutationTaster. The ACMG/AMP guidelines for the interpretation of sequence variants class the mutation as pathogenic (PVS1, PM2, PP1).

## Discussion

The intellectual disability type 5 (MRT5) is a rare autosomal recessive disorder. In 2012, Khan et al. provided the first report of mutations in the *NSUN2* gene of three unrelated, affected individuals with neurodevelopmental disability, microcephaly, short stature and growth restriction [7]. A total of 30 patients with NSUN2-intellectual disability syndrome have since been reported [6–17]. The clinical presentation of these subjects with NSUN2 deficiency includes facial dysmorphism, microcephaly, short stature, ID, growth restriction, language impairment, hypotonia and delayed puberty. In the current study, we performed WES and identified a homozygous frameshift variant in the *NSUN2* gene in two Chinese patients.

We detected a homozygous pathogenic c.1171_1175delACCAT(p.Thr391fs*18) mutation in the eleventh exon of the *NSUN2* gene in our two patients. The deletion is a novel frameshift variant that causes a premature termination codon and may act similarly to other LoF variants in *NSUN2*, such as frameshifts, nonsense variants and splice sites. Previously reported LoF *NSUN2* variants include c.538-1G>C, c.1020delA(p.Gly341Valfs*15), c.679C>T(p.Gln227*) and c.538-11T>G(p.Ile179Argfs*192). These variants may result in an absence of protein production and markedly reduced mRNA levels, due to NMD degradation [6, 8, 10]. In accordance with the ACMG/AMP standards and guidelines, the c.1171_1175delACCAT(p.Thr391fs*18) variant is pathogenic, using the PVS1, PM2 and PP1 criteria.

Our patients showed the common phenotypes associated with MRT5. These included facial deformities (long face, ptosis, long palpebral fissures, high posterior hairline and smooth philtrum), moderate ID, microcephaly, short stature and language impairment. In addition, other physical complications, such as seizures, myelination delay and hearing impairment, were observed in our patients. Of interesting, the NSUN2 mutation in patient 1 and patient 2 was identical but the patients had different clinical presentations. Patient 1 had a history of epilepsy, which was controlled, from four years of age, with VPA. However, delayed myelination and moderate hearing impairment were observed in patient 2. Phenotypic heterogeneity of performance may be caused by individual differences of the patients.

To analyze the genotype and phenotype correlations in individuals with NSUN2 variants, the clinical and molecular features of the 30 patients and our two patients were documented and summarized (Table S1). The phenotypes of MRT5 are complex but several common characteristics are observed in most patients. All 32 patients had an abnormal neurodevelopmental phenotype, which ranged from moderate to severe ID. Dysmorphic features, which become more characteristic with age, were observed in all patients. They included long face, large/long nose with high nasal bridge, smooth philtrum, downturned upper lip and ocular abnormalities. Motor delays or impairment were also observed in all patients (17/17). All of them learned to walk, although not until after the age of six in some cases. Hypotonia was common (19/32). Three patients had an ataxic gait, whilst two other patients had a broad gait. Language was generally affected (22/22) and ranged from delayed speech to remaining nonverbal in adulthood. Of the 11 patients that were six years of age or older, ten were nonverbal. Short stature (26/29) and microcephaly (25/27), which are two common features of the disease, were two to six standard deviations below the mean of the general population. Delayed puberty may be another common feature of the disease and was observed in eight of the 10 patients that were older than 10. Three of the affected women had impaired sexual development, which included hypoplastic breasts, no pubic hair, amenorrhea, uterine dysplasia and atrophied or absent ovaries. In addition, the affected men had testicular hypoplasia, cryptorchidism and reduced testosterone levels. A subset had feeding difficulties (8/12) and brain abnormalities were common (8/15). These included delayed myelination, cerebellar atrophy, hypomyelination, dysplastic corpus callous and simple frontal gyral pattern. Additionally, a number of variable manifestations were observed in these patients. Several patients (7/27) had skeletal or limb abnormalities. Six had seizures and six had hearing impairment. Other dysmorphic features, such as eczema, juvenile cataracts, chronic nephritis and growth hormone (GH) deficiency, were also observed.

A total of 17 variants were observed, of which loss-of-function variants (LoF; including non-sense variants, splicing variants and frameshifts ) were the most common type (Table S1)\[6–17]. We compared the phenotypes of five patients with homozygous missense variants and 26 patients with homozygous loss-of-function variants (Table 1). There appears to be a wider range of phenotypes in patients with LoF variants. Some phenotypes, such as brain abnormalities (8/15), hearing impairment (5/18), seizures (6/24), feeding issues (6/10) and other variable manifestations, including eczema, juvenile cataracts, chronic nephritis and growth hormone (GH) deficiency, have only been observed in patients carrying the LoF variants. However, it is important to note that even patients who carry the same LoF variant may exhibit diverse phenotypes. Neurodevelopmental disorders caused by the *NSUN2* gene exhibit a wide range of complex clinical symptoms. Comparing these symptoms between patients helps to improve our understanding of clinical spectrum of the disorder. Although some consistent clinical manifestations were observed, we currently cannot point to a clear genotype-phenotype correlation. The phenotypic variability observed in these patients may be attributed to the pleiotropic effects of the underlying variants. These results were based on observations from the current case and investigations into an increased number of patients are expected to further refine the phenotype, identify genotype effects and other phenotypic determinants.

**Table 1:** Comparison of effects of variant type on clinical manifestations

	Missense mutations	^a^LOF mutations
Motor delay or disability	5/5	14/14
Development of speech and language skills	5/5	16/16
Nonverbal	1/5	11/17
ID	5/5	26/26
Moderate to profound	2/5	8/25
Microcephaly	3/3	23/23
Short stature	4/4	21/24
Eye findings	2/3	12/15
Facial dysmorphism	5/5	26/26
Muscular hypertonia	3/3	16/22
Brain radiologic features	NR	8/15
Feeding issues	NR	6/10
Delayed puberty	1/3	7/7
Seizures	0/3	6/24
Skeletal or limb abnormalities	3/3	4/23
Hearing impairment	0/3	5/18

*Abbreviation: ID* Intellectual disability, *NR* Not reported, *LoF* Loss of function

^a^All deletions,duplication, frameshift, nonsense, start-loss, and splice site were considered predicted LoF for this table

It is unclear how these variants cause ID, facial dysmorphism, microcephaly, short stature, growth restriction, language impairment, hypotonia, delayed puberty and additional clinical symptoms. Multiple mechanisms have been hypothesized to explain NSUN2-related intellectual disability. The function of the *NSUN2* protein is to modify nucleotides at tRNA-leu (CAA) wobble positions and these modifications are required for stabilization of anticodon-codon pairings and for correct translation of mRNA pairings[19]. Loss of function of *NSUN2* may cause the absence or reduction of tRNA modification, which results in altered tissue-specific protein expression [20]. Another possible disease mechanism may involve the role of *NSUN2* in impaired methylation of hemi-methylated DNA. Changes in DNA methylation patterns can alter gene transcription patterns and promote mutations [21]. Furthermore, *NSUN2* is involved in cell cycle regulation, through the stabilization of the mitotic spindle, and functionally impaired *NSUN2* may lead to abnormal cell division [3, 4]. Further functional studies will contribute to our understanding of NSUN2-intellectual disability syndrome and the mechanisms of action.

## Conclusion

In summary, we uncovered a novel, homozygous, pathogenic variant in *NSUN2* in two Chinese patients, from the same family, with moderate ID, dysmorphic facies, microcephaly, short stature, DD, language impairment and other congenital abnormalities. This is the first report of the c.1171_1175delACCAT(p.Thr391fs*18) variant in the *NSUN2* gene and it will expand the mutation spectrum of NSUN2-related intellectual disability. The convergence and divergence of phenotypes caused by different variants within *NSUN2* suggests that further functional studies, to investigate the site-specific functions of different variants, could help to enhance our understanding of this disease and its mechanisms of action. The detailed genotype-phenotype correlations reported in the present study are important for the genetic diagnosis and accurate clinical management of patients with NSUN2-intellectual disability syndrome.

### Supplementary Information

**Supplementary Material 1. **

## Data Availability

The datasets presented in this study can be found in online repositories. The names of the repositories and accession numbers can be found at https://www.ncbi.nlm.nih.gov/sra/PRJNA865489.

## Abbreviations

MRT5: Intellectual disability type 5
ID: Intellectual disability
LOF: Loss-of-function
DD: Developmental delay
GTCS: Generalized tonic-clonic seizure
EEG: Electroencephalogram
VPA: Valproate
WES: Whole exome sequencing
SNV: Single nucleotide variants
NMD: Nonsense-mediated mRNA decay
GH: Growth hormone
